# Metabolomic response of *Perilla frutescens* leaves, an edible-medicinal herb, to acclimatize magnesium oversupply

**DOI:** 10.1371/journal.pone.0236813

**Published:** 2020-07-29

**Authors:** Ha In Mun, Yangmin X. Kim, Dong Ho Suh, Seulbi Lee, Digar Singh, Eun Sung Jung, Choong Hwan Lee, Jwakyung Sung

**Affiliations:** 1 Department of Bioscience and Biotechnology, Konkuk University, Seoul, Korea; 2 National Institute of Agricultural Sciences, Rural Development Administration, Wanju, Republic of Korea; 3 Department of Systems Biotechnology, Konkuk University, Seoul, Korea; 4 Research Institute for Bioactive-Metabolome Network, Konkuk University, Seoul, Korea; 5 Department of Crop Science, College of Agriculture, Life and Environment Sciences, Chungbuk National University, Cheongju, Korea; Institute for Biological Research "S. Stanković", University of Belgrade, SERBIA

## Abstract

High salt accumulation, resulting from the rampant use of chemical fertilizers in greenhouse cultivation, has deleterious effects on plant growth and crop yield. Herein, we delineated the effects of magnesium (Mg) oversupply on *Perilla frutescens* leaves, a traditional edible and medicinal herb used in East-Asian countries. Mg oversupply resulted in significantly higher chlorophyll content coupled with lower antioxidant activities and growth, suggesting a direct effect on subtle metabolomes. The relative abundance of bioactive phytochemicals, such as triterpenoids, flavonoids, and cinnamic acids, was lower in the Mg-oversupplied plants than in the control. Correlation analysis between plant phenotypes (plant height, total fresh weight of the shoot, leaf chlorophyll content, and leaf antioxidant content) and the altered metabolomes in *P*. *frutescens* leaves suggested an acclimatization mechanism to Mg oversupply. In conclusion, *P*. *frutescens* preferentially accumulated compatible solutes, *i*.*e*., carbohydrates and amino acids, to cope with higher environmental Mg levels, instead of employing secondary and antioxidative metabolism.

## Introduction

*Perilla frutescens* (L.) Britt., an herb belonging to the family Lamiaceae, is widely distributed in East-Asian countries, including Korea, China, and Japan, where it has been used as a culinary seasoning and in traditional medicine for centuries [1]. *P*. *frutescens* leaves are believed to possess antioxidant, anti-inflammatory, and anti-allergic properties, owing to the high abundance of bioactive phytochemicals, including polyphenolic compounds, triterpenoids, and anthocyanins, among others [2, 3]. These phytochemicals allow plants to overcome various biotic and abiotic stress conditions, including nutrient availability, temperature extremes, light intensity, and microbial attack, which can be pervasive in the plant’s micro-environment [4]. The abiotic stress conditions are the major environmental limiting factors in modern agricultural practices and are responsible for significant crop yield losses worldwide [4].

Intensive cultivation of crops in greenhouses coupled with the rampant application of chemical fertilizers often results in the accumulation of salts in the soil, leading to nutrient imbalances [5]. High salinity can alter various physiological and biochemical processes that protect against secondary stress conditions, such as oxidative stresses, osmotic imbalance, and water and nutrient deficits, thus adversely affecting plant growth. In recent years, several studies have described the various minerals associated with salt accumulation, including magnesium (Mg^2+^) and sodium (Na^+^) [6–8]. Furthermore, magnesium (Mg) is an essential macronutrient playing a key role in plant growth and development [9]. It is the most abundant free divalent cation in the cytosol, and, in particular, Mg plays a central role in plant chlorophyll biosynthesis and carbon fixation as a cofactor of a series of enzymes involved in carbon metabolism [10]. However, the importance of Mg in crop production was underestimated in the last decades. Indeed, Mg deficiency is typically correlated with visible interveinal chlorosis and growth reduction, and yield of crops [11]. However, both the Mg oversupply as well as the deficiency results in stress conditions which varyingly affects plant morphogenesis, development, and yield through various mechanisms. Especially, the role of Mg induced stressed conditions on stimulating the plant stress response involving the phytohormones, downstream metabolism (C-fixation), ion uptake and antioxidant mechanisms is largely unknown [12]. For these causes, it is crucial to examine the effects of Mg oversupply in plants.

‘Metabolomics’ is being increasingly applied to investigate plant physiological responses to different abiotic stress conditions, including drought, salinity, and temperature extremes [13]. As the final downstream product of the genome, the metabolome is composed of numerous small-sized molecules (≤ 2000 Da) mediating almost every biomolecular mechanism, including stress tolerance and the response to environmental cues [14]. Thus, metabolomics represents an important tool for investigating plant stress response mechanisms under certain environmental conditions [15]. Herein, we examined the untargeted MS-based metabolomic profiles of *P*. *frutescens* leaves in plants oversupplied with Mg, using high throughput GC-MS and LC-MS platforms and multivariate analyses. This study aimed to delineate the metabolic response mechanisms of *P*. *frutescens* following Mg oversupply under controlled conditions.

## Materials and methods

### Chemicals and reagents

Methanol, ethanol, acetonitrile, and water were purchased from Fisher Scientific (Waltham, MA, USA). Trolox, methoxyamine hydrochloride, pyridine, N-methyl-N-(trimethylsilyl) trifluoroacetamide (MSTFA), 6-hydroxy-2,5,7,8-tetramethylchroman-2-carboxylic acid, hydrochloric acid, potassium persulfate, 2,2′-azinobis (3-ethylbenzothiazoline-6-sulfonic acid) diammonium salt (ABTS), hydrochloric acid, 2,4,6- tris(2-pyridyl)-trizine (TPTZ), iron(III) chloride hexahydrate, sodium acetate, acetic acid, sodium carbonate, sodium hydroxide, 1,1-diphenyl-2-picrylhydrazyl (DPPH), formic acid, and the standard compounds were obtained from Sigma Chemical Co. (St. Louis, MO, USA).

### Plant growth, mineral supply, and the measurement of physiological parameters

One-month-old *Perilla frutescens* seedlings (*Perilla frutescens* var. *japonica*, cv. Sangyeop) were transplanted into plastic pots (3.6 L) containing soil on September 11, 2018. They were grown for 13 weeks in a greenhouse at the National Institute of Agricultural Sciences, RDA, in Jeonju, Korea. The temperature was maintained between 15–35°C, with daily fluctuations. Plants were grown under the natural sunlight and the greenhouse roof was shielded with a layer of curtain to prevent a high light intensity, which may cause adverse effects on growth of *P*. *frutescens* in Mg excess soil conditions. Following the natural sunlight exposure, the plants were subjected to extended photoperiod through artificial illumination using lamps was applied from 19:00 to 22:00 hours using lamps. To investigate the effects of varying titers of Mg supply, the initial concentration of the fertilizer was adjusted so that the Mg concentration was either 7.5 (M7.5) or 10 (M10) times compared to the control, before transplanting. The soil magnesium concentrations at the end of harvest were 3.5, 5.9, and 9.3 cmol.kg^-1^ for the control, M7.5, and M10 groups, respectively. The soil Mg concentration was analyzed with an inductively coupled plasma optical emission spectrometry (ICP-OES; Integra XL, GBC, Braeside, Australia). Soil electrical conductivity (EC) lower than 2.0 dS.m^-1^ is suitable for growing *Perilla frutescens* in a greenhouse; the M7.5 and M10 Mg oversupply treatments were characterized by slightly higher and considerably higher EC values than 2.0 dS.m^-1^, respectively. The soil EC values in the control, M7.5, and M10 groups were 0.9, 2.6, and 6.3 dS.m^-1^, respectively. The plants were watered at the rate of soil water potential of 30 kPa.day^-1^ in the early growth stages and 60 kPa.day^-1^ between four to thirteen weeks of cultivation. The soil water potential was measured by a tensiometer (Soilmoisture Equipment Corp., Santa Babara, USA), whose gypsum block that senses the water potential was placed at depth of the 0.06–0.12 m below the soil surface in the pot. At the first harvest, old leaves up to the fourth node for plants in the M7.5 and M10 groups and up to the fifth node for plants in the control group were weighed using an electronic balance and then discarded. To evaluate the physiological parameters at final harvest, the plant height was recorded, and the leaf chlorophyll content was evaluated using a chlorophyll meter (SPAD-502Plus, Konica Minolta Sensing, Osaka, Japan) from three different plants in each treatment group. Chlorophyll meter measured three leaves from each node (5^th^ to 7^th^ node for M7.5 and M10 groups; 6^th^ to 8^th^ node for the control) and their average value was used as a leaf chlorophyll content. At the final harvest, a stem and six fully-expanded leaves between the fifth and seventh nodes were harvested in the M7.5 and M10 groups, and a stem and six fully-expanded leaves between the sixth and eighth nodes were harvested in the control group. Samples were harvested at 10:00. The leaf and stem samples at the final harvest were weighed using an electronic balance; these weights were then added to the weights at the first harvest to obtain the total fresh weight (g.plant^-1^). Harvested leaves were rinsed with distilled water and the distilled water was removed. Harvested leaves from a plant were sealed in a plastic bag and stored at -80°C. All analyses of metabolites were performed for three biological replicates obtained from three different plants in each treatment group.

### Sample harvest and extraction

The freeze-dried *P*. *frutescens* leaves were pulverized using liquid nitrogen, mortar, and pestle. Each powdered sample (100 mg) was extracted twice with 1 mL of mixed solvent (ethanol/water, 70/30, v/v) containing 5 μL of internal standard (0.5 mg.mL^-1^ 2-chlorophenylalanine), using a MM400 mixer-mill (Retsch^®^; Haan, Germany) at a frequency of 30 s^-1^ for 10 min. The extract was sonicated for 10 min and centrifuged at 17,000 rpm for 10 min at 4°C (Hettich Zentrifugen Universal 320, Tuttlingen, Germany). The supernatant was collected into an e-tube and filtered through a 0.2-μm polytetrafluoroethylene (PTFE) filter, and then completely dried using a speed vacuum concentrator (Modulspin 31; BioTron, Inc., Bucheon-si, Korea). The dried samples were used for instrument analyses and bioactivity assays.

### GC-TOF-MS analysis

For derivatization, 100 μL of the supernatant was transferred into a fresh e-tube and dried completely. For the oximation step, 50 μL of methoxyamine hydrochloride (20 mg.mL^-1^ in pyridine) was added to the dried extracts, and the reaction was carried for 90 min at 30 °C. For the silylation step, 50 μL of MSTFA was added to the reaction, and the mixture was incubated at 37 °C for 30 min. The samples were then filtered, and the final concentration of the derivatized samples was reconstituted to 10 mg.mL^-1^. The quality control (QC) sample was prepared by pooling 10 μL aliquots from all samples.

The gas chromatography-time of flight-mass spectrometry (GC-TOF-MS) analysis was performed on an Agilent 7890A GC system (Agilent Technologies, Palo Alto, CA, USA) coupled with an Agilent 7693 autosampler (Agilent Technologies) and Pegasus HT TOF MS (LECO, St. Joseph, MI, USA) system. Chromatographic separation was achieved on an Rtx-5MS column (30 m length × 0.25 mm inner diameter; J & W Scientific, USA), with a constant flow of 1.5 mL.min^-1^ of helium as the carrier gas. The analytical program employed for sample analysis was adopted from a previous study [16]. We maintained three biological replicates of each sample, and analyses were performed in a random order to minimize bias.

### LC-MS analysis

The dried extracts were reconstituted in appropriate solvents for ultra-high-performance liquid chromatography linear trap quadrupole orbitrap tandem mass spectrometry (UHPLC-LTQ-Orbitrap-MS/MS) analysis. The final concentration of all the analytes was 20 mg.mL^-1^. The UHPLC system was equipped with a Vanquish binary pump H system (Thermo Fisher Scientific, Waltham, MA, USA) coupled with an auto-sampler and a column compartment. Chromatographic separation was achieved on a Phenomenex KINETEX^®^ C18 column (100 mm × 2.1 mm, 1.7 μm particle size; Torrance, CA, USA), with a sample injection volume of 5 μL. The MS data were collected in the range of 100–1500 m/z (under negative- and positive-ion modes) using an Orbitrap Velos Pro^™^ system, which was combined with an ion-trap mass spectrometer (Thermo Fisher Scientific) coupled with a HESI-II probe. The samples were analyzed following the protocols described by Kwon et al. [17].

The UV absorbance values of the analytes were compared against their corresponding peaks using ultra-high-performance liquid chromatography linear trap quadrupole tandem mass spectrometry (UHPLC-LTQ-ESI-IT-MS/MS). The system consisted of a DIONEX UHPLC system that included an UltiMate 3000 RS pump, UltiMate 3000 RS autosampler, UltiMate 3000 column compartment, and UltiMate 3000 variable wavelength detector (Dionex Corporation, Sunnyvale, CA, USA), coupled with an LTQ XL^™^ ion-trap mass spectrometer (Thermo Fisher Scientific). Chromatographic separation was achieved on a Syncronis C18 column (100 mm × 2.1 mm, 1.7 μm particle size; Thermo Fisher Scientific), with an injection volume of 10 μL. The program for sample analysis was adopted from a previous study [16].

### Data processing and multivariate statistical analysis

Raw datasets generated from the GC-TOF-MS analysis were converted to the Net CDF format (*.cdf) using LECO Chroma TOF software (version 4.44, LECO Corp.). The UHPLC-LTQ-Orbitrap-MS/MS data were acquired using Xcalibur software (version 2.00, Thermo Fisher Scientific) and converted into the Net CDF format (*.cdf). The CDF files were preprocessed using the MetAlign software package (http://www.metalign.nl) for peak detection, retention time correction, and alignment. The resulting data were exported to an Excel spreadsheet (Microsoft; Redmond, WA, USA). Multivariate statistical analyses were performed using SIMCA-P+ software (Ver. 12.0, Umetrics; Umeå, Sweden). The datasets were auto-scaled (unit variance scaling) and mean-centered in a column-wise manner. The significantly different metabolites between the treated (Mg-oversupplied) and control *P*. *frutescens* samples were selected based on the VIP values obtained from the orthogonal projection to latent structures-discriminant analysis (OPLS-DA) score plot, and significant differences (*p* < 0.05) between experimental groups were tested using analysis of variance (ANOVA) and Duncan’s multiple range tests, performed using PASW Statistics 18 software (SPSS, Inc., Chicago, IL, USA). Significantly different metabolites were tentatively identified by comparing their retention times, mass fragment patterns, and elemental compositions derived from the UHPLC-LTQ-Orbitrap-MS/MS analyses with standard compounds, in-house library, and published literature. The correlation map was obtained using PASW Statistics 18.0 software (SPSS Inc., Chicago, IL, USA) and constructed using MeV software (http://www.tm4.org/).

### Antioxidant activity assays

Three antioxidant activity tests were performed, *i*.*e*., 2,2’ -azino-bis(3-ethylbenzothiazoline-6-sulfonic acid (ABTS), 1,1-diphenyl-2- picrylhydrazyl (DPPH), and ferric reduction ability of plasma (FRAP), using previously described procedures with slight modifications [18]. The final concentration of the dried sample extracts was reconstituted to 0.5 mg/mL by adding 70% EtOH for these assays. For the ABTS assay, 10 μL of the sample extract was added to 190 μL of the diluted ABTS solution (O.D. ~ 0.7 at 750 nm) in a 96-well plate, and the reaction was incubated in the dark for 6 min before measuring the absorbance at 734 nm using a microplate reader (Spectra MAX190, Molecular Devices, San José, CA, USA). For the DPPH assay, 20 μL of the sample extract was mixed with 180 μL of 0.2 mM DPPH ethanol solution in a 96-well plate, and the reaction was incubated for 20 min at room temperature, following which the absorbance of the mixture was recorded at 515 nm. For the FRAP assay, 10 μL of the sample extract was mixed with 300 μL of the FRAP reagent, and the reaction mixture was incubated for 6 min at 37 °C in 96-well microtiter plates. The absorbance was measured at 570 nm using a microplate reader. The results of all the assays were presented as the Trolox equivalent antioxidant capacity (mM), with the standard solution concentration curve ranging from 0.0078 to 1.000 mM. All assays were performed with three biological and analytical replicates.

## Results

### Mg oversupply affects plant growth and leaf antioxidant content in *P*. *frutescens*

**Fig 1:**
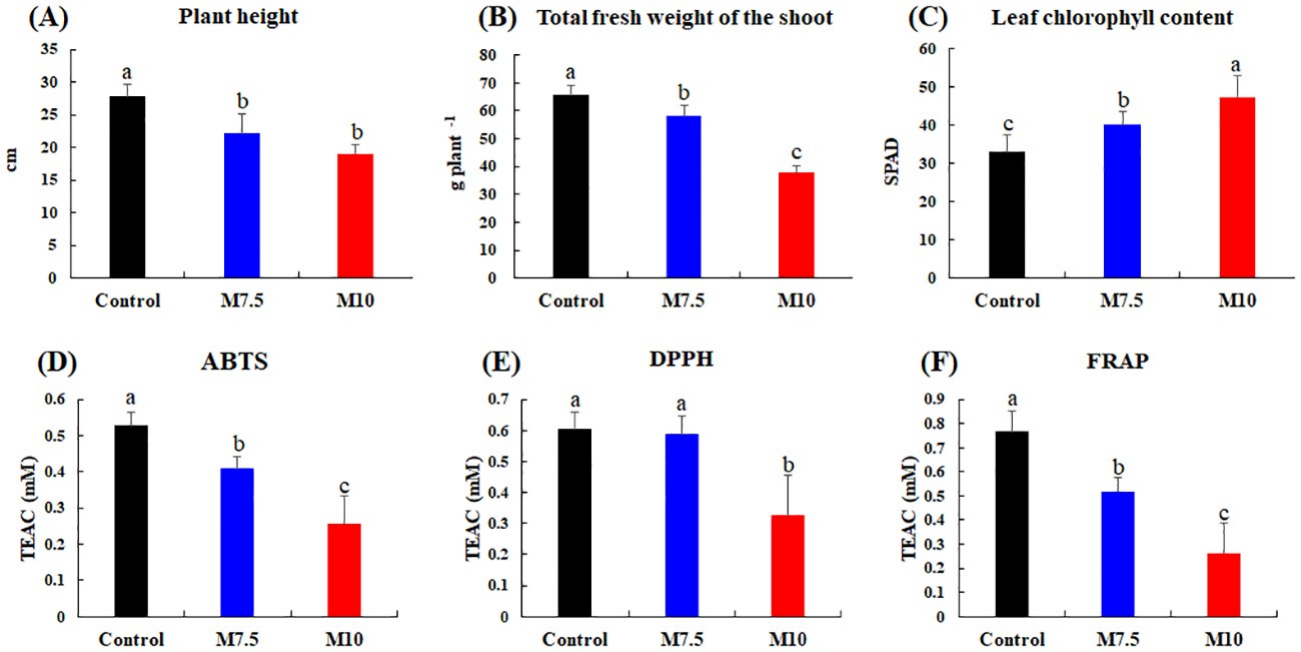
Plant height (A), total fresh weight of the shoot (B), leaf chlorophyll contents (C), and leaf antioxidant activities [ABTS (D), DPPH (E), and FRAP (F)] of *Perilla frutescens* leaves under control and magnesium oversupply. Different letters over the bars in the graphs indicate statistically significant differences based on Duncan’s multiple range test (*p*-value < 0.05). TEAC: Trolox equivalent antioxidant capacity. M7.5, magnesium supply at 7.5 times the control; M10, magnesium supply at 10 times the control. Three different plants were used as biological replicates.

We categorically examined plant growth characteristics (plant height, total fresh weight of the shoot, and leaf chlorophyll content) and leaf antioxidant activities (2,2’ -azino-bis(3-ethylbenzothiazoline-6-sulfonic acid (ABTS), 1,1-diphenyl-2- picrylhydrazyl (DPPH), and ferric reduction ability of plasma (FRAP)) in *P*. *frutescens* subjected to Mg oversupply at two different levels, M7.5 and M10 (Fig 1). The plant height and total fresh weight of the shoot decreased linearly with increasing Mg supply in the following order; Control > M7.5 > M10, at *p* < 0.05 (Fig 1A and 1B). In contrast, the leaf chlorophyll content increased with increasing Mg supply, as shown in Fig 1C. The antioxidant activities (ABTS, DPPH, and FRAP) in the *P*. *frutescens* leaves decreased uniformly with increasing Mg supply (Fig 1D–1F), with the lowest leaf antioxidant activities observed in the plants subjected to the M10 treatment (Fig 1D–1F).

### Mg oversupply alters the metabolite profiles in *P*. *frutescens* leaves

**Fig 2:**
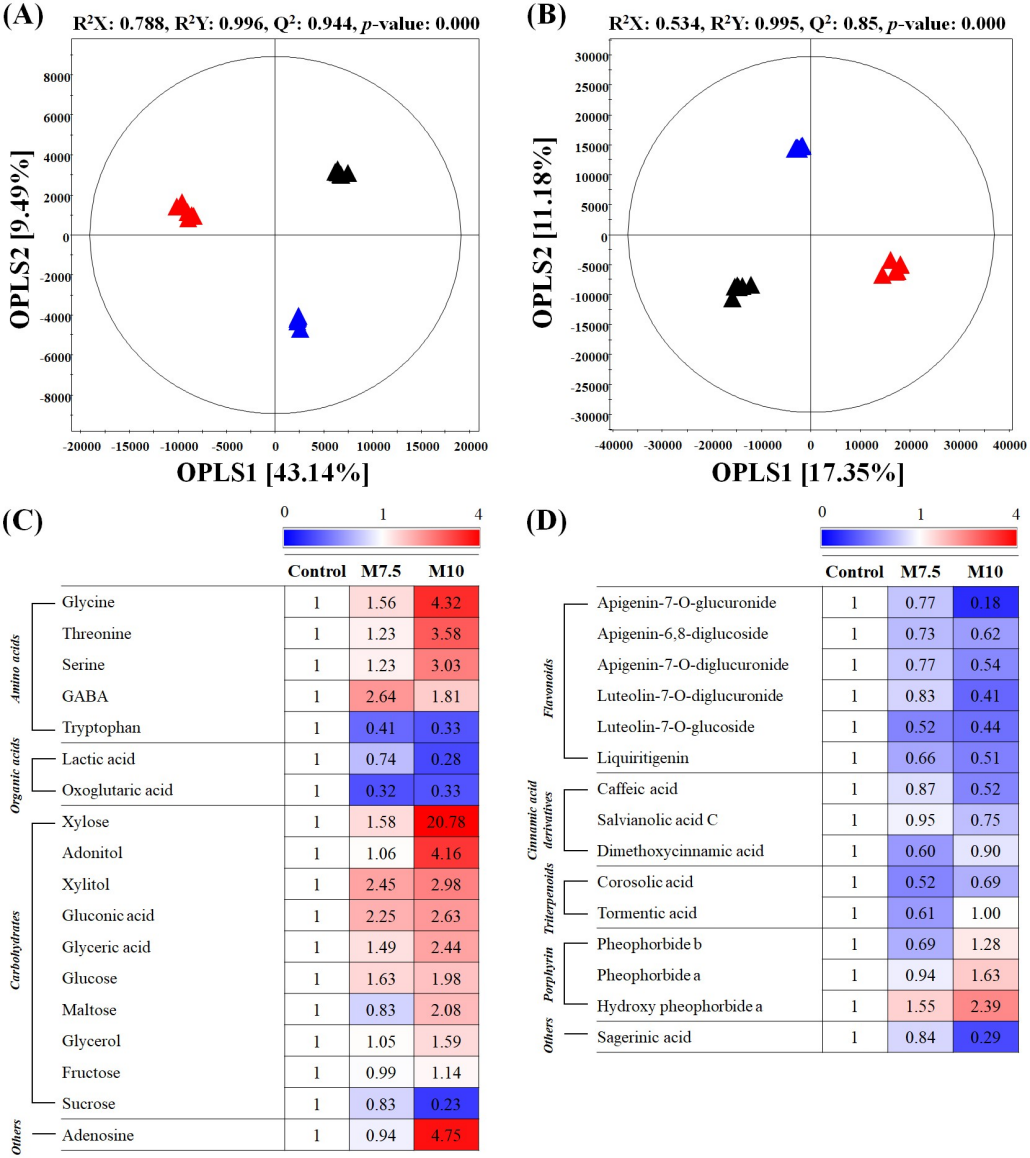
Orthogonal projection to latent structures-discriminant analysis (OPLS-DA)) score plots of *Perilla frutescens* leaves under control and magnesium oversupply derived from GC-TOF-MS (A) and UHPLC-LTQ-Orbitrap-ESI-MS/MS (B). Heat map analysis of *P*. *frutescens* leaves under control and magnesium oversupply based on GC-TOF-MS (C) and UHPLC-LTQ-Orbitrap-ESI-MS/MS (D) data. The heat map represents the relative abundance of significantly discriminant primary and secondary metabolites (VIP >0.7, *p*-value <0.05) based on the OPLS-DA model. The relative content is presented as fold-changes normalized by the control value. Symbols indicated: ▲, Control; ▲, M7.5; ▲, M10. M7.5, magnesium supply at 7.5 times the control; M10, magnesium supply at 10 times the control. Three different plants were used as biological replicates.

In congruence with the phenotype data (Fig 1), the untargeted metabolite profiles of *P*. *frutescens* leaf extracts exhibited considerable variations following the different Mg treatments. The principal components analysis (PCA) score plot based on the GC-TOF-MS datasets presented a clustered pattern, with the M10 group segregated from the control and M7.5 groups across PC1 (S1A Fig). The class-wise variance among the metabolite profiles of three *P*. *frutescens* leaves groups (control, M7.5, M10) were highlighted using the supervised orthogonal projection to latent structures-discriminant analysis (OPLS-DA) score plot. The OPLS-DA score plot showed a similar pattern, with the metabolomic datasets from the M10 group segregated from the remaining datasets (M7.5 and Control) across OPLS1 (28.65%) and the control group clustered separately from the M7.5 group across OPLS2 (12.25%), as shown in Fig 2A. The OPLS-DA validation was evident from the satisfactory model parameters, including R^2^X (0.593), R^2^Y (0.995), Q^2^ (0.947), and *p*-value (0.004). The PCA score plot based on the UHPLC-LTQ-Orbitrap-MS/MS datasets presented a clustered pattern, with the M10 group segregated from the control and M7.5 groups across PC1 (S1B Fig). The OPLS-DA score plot based on the UHPLC–LTQ-Orbitrap-MS/MS (Fig 2B) data displayed a distinct clustering pattern, highlighting the metabolomic disparity among the Mg-treated (M10 and M7.5) and untreated plant groups. Notably, all three groups were distinctly segregated across OPLS1 (20.93%) and OPLS2 (12.57%), with model parameters R^2^X (0.472), R^2^Y (0.996), and Q^2^ (0.922) with a *p*-value of 0.000.

We selected 34 significantly discriminant primary and secondary metabolites (S1 and S2 Tables) that were significantly different between the leaf extracts of the Mg-treated (M7.5 and M10) and control groups, based on the OPLS-DA models (Fig 2A and 2B) with variable importance in projection (VIP) values >0.7. These discriminant metabolites represented different compound classes, namely amino acids, carbohydrates, fatty acids, organic acids, flavonoids, cinnamic acid derivatives, triterpenoids, and chlorins, among others. The relative levels of the discriminant metabolites in the *P*. *frutescens* leaves under Mg oversupply were visualized as a heat map based on the VIP values (>0.7) and *p*-value (<0.05) (Fig 2C and 2D). We observed a gradual increase in the relative abundance of amino acids (glycine, threonine, serine, and GABA), carbohydrates (xylose, adonitol, xylitol, gluconic acid, glyceric acid, glucose, maltose, glycerol, and fructose), and chlorins (pheophorbide b, pheophorbide a, and hydroxy pheophorbide a) in the Mg-treated groups, compared with the control. In contrast, we observed a decrease in the relative content of tryptophan, some organic acids (lactic acid and oxoglutaric acid), sucrose, flavonoids (apigenin-7-O-glucuronide, apigenin-6,8-diglucoside, apigenin-7-O-diglucuronide, luteolin-7-O-diglucuronoide, luteolin-7-O-glucoside, and liquiritigenin), cinnamic acid derivatives (caffeic acid, salvianolic acid C, and dimethoxycinnamic acid), triterpenoids (corosolic acid and tormentic acid), and sagerinic acid in the Mg-treated groups, compared with the control.

### Correlations between the metabolite profile and the plant phenotypes of *P*. *frutescens* following Mg oversupply

**Fig 3:**
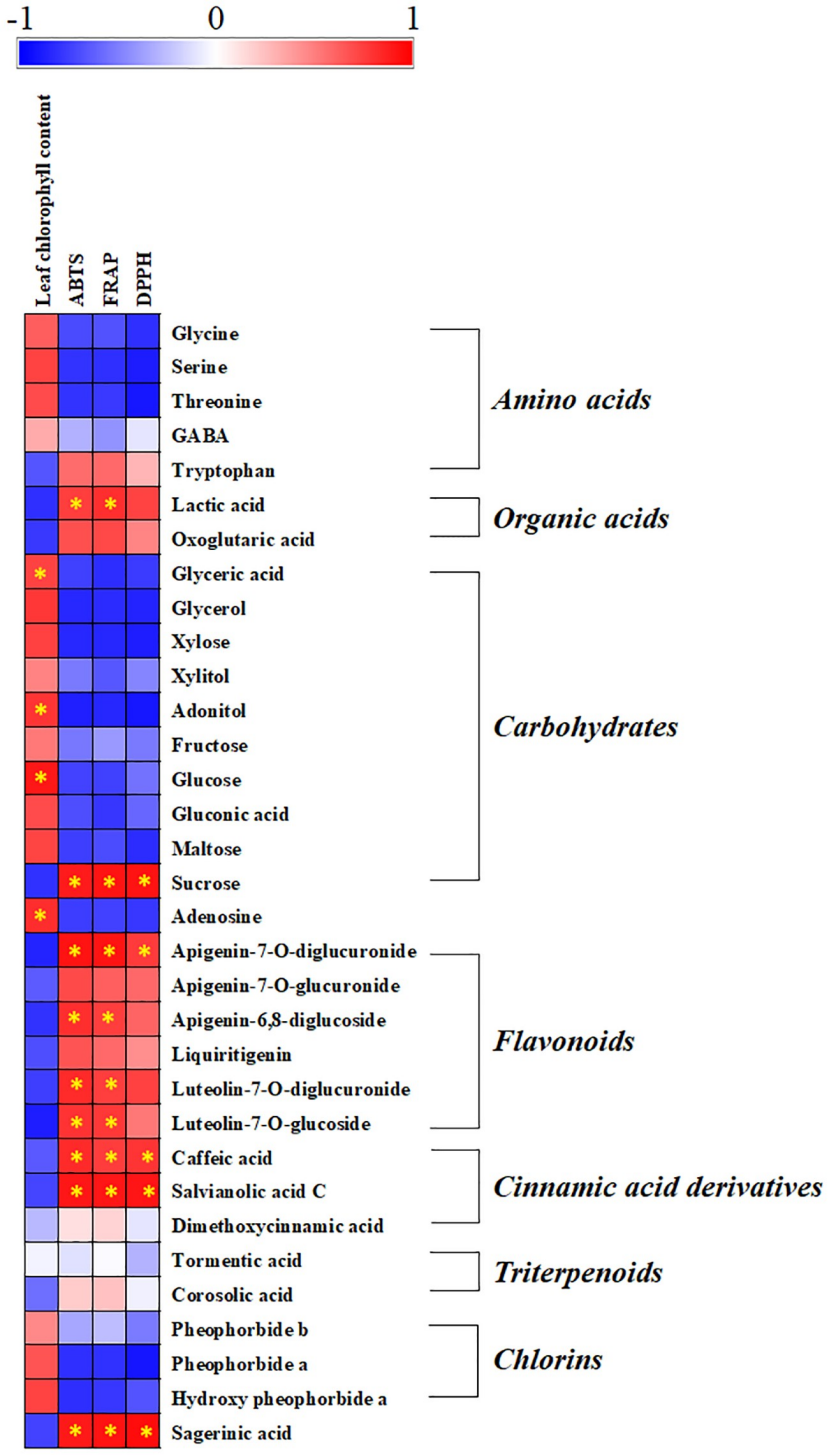
A correlation map for the significantly different metabolites and physicochemical parameters (leaf chlorophyll content, ABTS, FRAP, and DPPH) of *Perilla frutescens* under control and magnesium oversupply. Each square indicates the Pearson’s correlation coefficient values (r) for a pair of metabolites and physicochemical parameters. The red color indicates positive (0 < r < 1) correlation, and the blue color indicates negative (−1 < r < 0) correlation. The asterisks indicate strong positive correlations between metabolites and physicochemical parameters (r >0.75).

We performed a correlation map analysis to visualize the correlations between the significantly altered metabolites and physicochemical data. It was evident from the correlation map (Fig 3) that plant height was strongly positively correlated (r > 0.75) with tryptophan, apigenin-7-*O*-diglucuronide, apigenin-6,8-diglucoside, and luteolin-7-*O*-glucoside. Total fresh weight of the shoot, ABTS, and FRAP exhibited a strong positive correlation (r > 0.75) with lactic acid, sucrose, apigenin-7-*O*-diglucuronide, apigenin-6,8-diglucoside, luteolin-7-*O*-diglucuronide, luteolin-7-*O*-glucoside, caffeic acid, salvianolic acid C, and sagerinic acid. Furthermore, the leaf chlorophyll content was positively correlated with most amino acids, carbohydrates, and chlorins and exhibited a strong positive correlation (r > 0.75) with glycerol, adonitol, glucose, and adenosine. To further understand the metabolomic response of *P*. *frutescens* leaves to Mg oversupply, we correlated the metabolic pathways of the significantly altered metabolites under high Mg stress with the identified metabolites that are involved in *P*. *frutescens* leaf homeostasis under magnesium oversupply.

## Discussion

High salt accumulation and nutrient imbalance can occur owing to the year-round greenhouse cultivation coupled with the excessive application of chemical fertilizers [6]. Salt stress can significantly influence plant growth and development by affecting the associated biochemical processes and metabolic pathways [19]. Although several studies have described the effects of high salinity on plants, the mechanisms correlating high Mg stress with plant growth phenotypes and metabolism are not fully understood. In the present study, we performed non-targeted metabolite profiling of *P*. *frutescens* leaf extracts to understand the metabolic changes that occur following Mg oversupply.

Regarding the effects of Mg oversupply on plant growth phenotypes (Fig 1A–1C), the highest titer of magnesium (M10) was associated with the lowest plant height and total fresh weight of the shoot. High magnesium titers have previously been reported to reduce plant biomass in barley [20]. Excess Mg in the cytoplasm may inhibit biochemical wall loosening and interfere with the uptake of other nutrients by the plant, such as Zn or Mn, thereby restricting plant growth [21]. Unlike the growth phenotypes, the leaf chlorophyll content of the plants in the M10 group was significantly higher than that in the M7.5 and control groups, which was consistent with the results of a previous study, which reported an increase in the chlorophyll content of strawberry leaves following treatment with Mg(NO_3_)_2_, because Mg ions are at the center of chlorophyll molecules [22]. In the case of the antioxidant activities (ABTS, DPPH, and FRAP), we observed the lowest bioactivities in leaf extracts from the plants subjected to the M10 treatment (Fig 1D–1F). In general, plants display higher antioxidant activities under stress conditions involving ROS [23]. However, the results of the antioxidant activity assays of the Mg-treated *P*. *frutescens* leaves obtained in this study were contradictory to those reported in previous studies.

**Fig 4:**
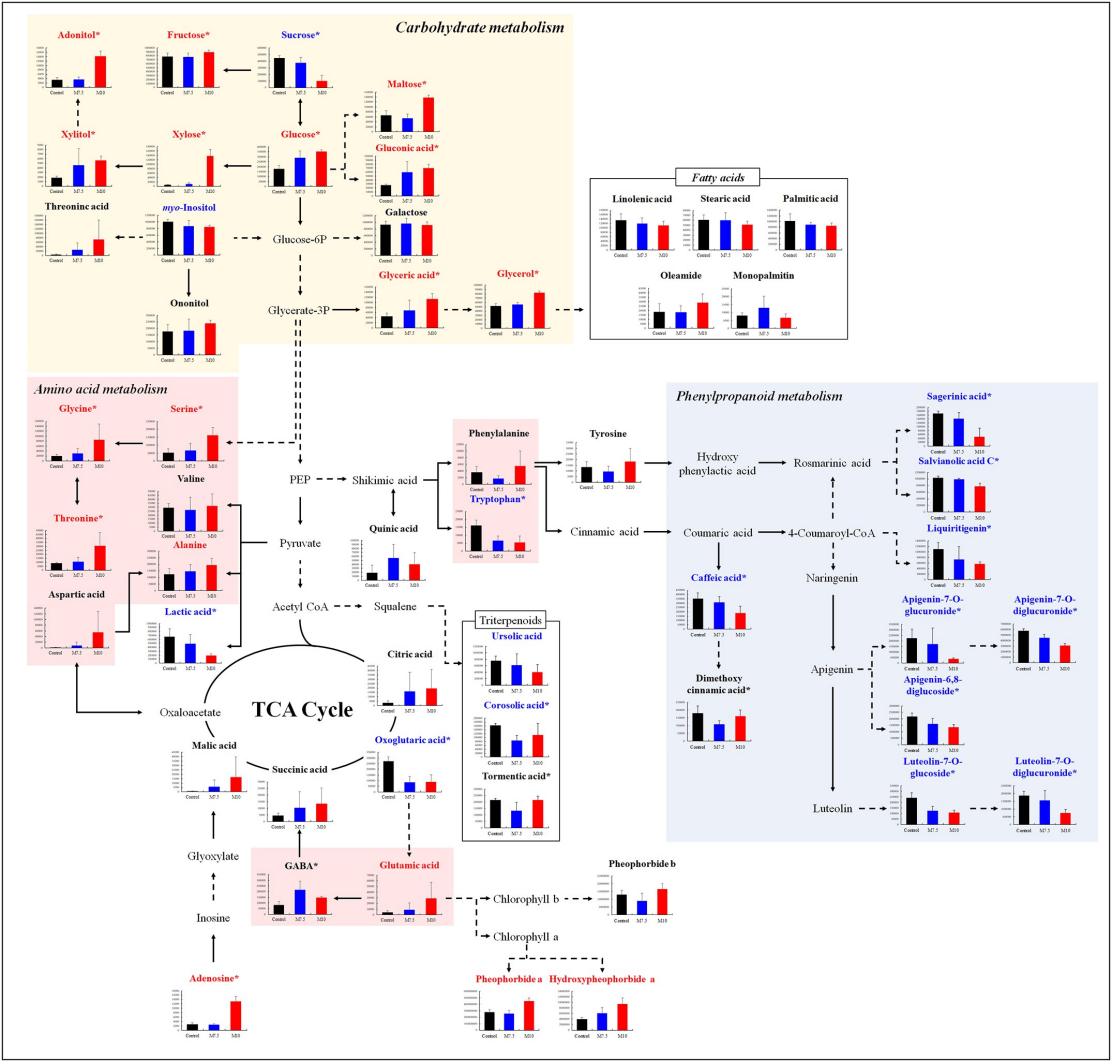
Schematic diagram of the metabolic pathway and relative content of metabolites in *Perilla frutescens* leaves under control and magnesium oversupply. The red and blue letters represent an increase and decrease in the levels of the metabolites in the M10 group compared to the control, respectively. Data are the mean values of the control, M7.5, and M10 groups, with the error bars indicating standard deviation. The metabolic pathway was modified from the KEGG database (http://www.genome.jp/kegg/). The asterisks indicate significantly different metabolites (VIP >0.7, p-value <0.05). M7.5, magnesium supply at 7.5 times the control; M10, magnesium supply at 10 times the control.

To determine the potential biological events triggered after Mg oversupply, we visualized the metabolic pathways involving the significantly discriminant metabolites in the *P*. *frutescens* leaf extracts (Fig 4). Based on the high relative abundance of most sugar derivatives and organic acids, we conjecture that Mg oversupply may have upregulated carbohydrate metabolism and the tricarboxylic acid (TCA) cycle in the *P*. *frutescens* leaves. Similarly, the relatively high levels of amino acids, including aromatic amino acids such as phenylalanine and tyrosine, could be attributed to the upregulated shikimate pathway in the leaves following Mg oversupply. A variation in the primary metabolite levels represents altered regulatory processes in plant cells; carbohydrates and amino acids are considered the key components of various regulatory metabolic reactions occurring during these processes. For example, the increased accumulation of compatible solutes (sugar derivatives, nitrogenous compounds, and organic acids) mitigates the effects of osmotic stress, by balancing the cytoplasmic osmotic potential to cope with high salt accumulation in the vacuoles and extracellular space, stabilizing the lipid membranes, and preventing the denaturation of proteins and enzymes [7, 24]. Hence, an abundance of compatible solutes, such as carbohydrates and amino acids, allows the plant to overcome high salinity stress, without affecting normal cellular metabolism and by maintaining low levels of ROS production.

Many studies have shown that carbohydrate metabolism plays a vital role in abiotic stress tolerance. Sugar derivatives, such as sorbitol, mannitol, glycerol, and pinitol, have been reported to protect biological macromolecules from the damaging effects of high salinity [25]. In this study, we observed the increased accumulation of most carbohydrates (xylose, adonitol, xylitol, gluconic acid, glyceric acid, glucose, maltose, glycerol, and fructose) in the plants treated with excessive Mg, except sucrose and myo-inositol, which exhibited decreased levels. Low levels of sucrose and starch, but high levels of reducing sugars, in NaCl-stressed grapevine leaves [26]. The increased levels of reducing sugars may have resulted from hydrolytic processes in the salt-affected leaves [26]. Moreover, the decrease in starch and sucrose and the increase in hexoses in barley suggested that sucrose hydrolysis may provide sugars for osmoprotection under osmotic stress [27]. Similarly, excess Mg may trigger a competition between mono- and di- or poly-saccharides, leading to an increase in monosaccharides, which is favorable for plant growth under osmotic stress. The increase in maltose observed in this study may have resulted from the hydrolysis of starch. Considering the carbohydrate metabolism, our observation was in line with the accumulation of sugar derivatives, which may be attributed to the plant’s adaptive response to maintain cellular osmolarity and energy metabolism [28].

Another class of primary metabolites, *i*.*e*., the amino acids, was observed to be more abundant in the *P*. *frutescens* leaf extracts from the M10 treatment group than in that from the M7.5 and control groups. We argue that some amino acids may also function as compatible solutes to maintain osmotic homeostasis following excessive Mg application. Certain N-containing compounds, including amino acids, have been reported to accumulate in plants under salt stress [29]. In addition, N-metabolism in plants is significantly altered under abiotic stress conditions, leading to the increased accumulation of amino acids owing to the increase in protein degradation to protein synthesis ratios [30, 31]. In the present study, we observed a significantly higher abundance of amino acids, including alanine, valine, threonine, aspartic acid, serine, glycine, tyrosine, phenylalanine, glutamic acid, and GABA, in leaf extracts from the M10 group than in those from the control. Since glutamic acid acts as a precursor of chlorophyll a and chlorophyll b, we carefully conjecture that the high abundance of glutamic acid in the M10 group may have resulted in the high chlorophyll content in the *P*. *frutescens* leaves (Figs 1C and 2C) [32]. Further, amino acids may act as precursors in the biosynthesis of secondary metabolites [33]. Aromatic amino acids, such as tyrosine and phenylalanine, act as intermediates in the shikimate pathway [34]. Hence, the high content of phenylalanine and tyrosine in the leaf extracts from the M10 group may be attributed to the regulation of stress-related pathways. However, it is worth mentioning that the amino acid levels may also increase owing to a reduction in growth as well as due to the stress response [7, 35].

This study demonstrates that Mg oversupply negatively affects the biosynthesis of functional metabolites, including flavonoids, cinnamic acids, triterpenoids, and sagerinic acid, in *P*. *frutescens* leaves, which may subsequently affect their bioactivity phenotypes. Considering the shikimate pathway, the levels of phenylpropanoid metabolism intermediates and cinnamic acid derivatives were surprisingly lower in the M10 group than in the M7.5 and control groups. This result agreed with previous results showing that phenolic compounds content in grapevine root stock decreases when the plants are subjected to Mg deficiency treatments because of main down-regulation of transcripts encoding for enzymes involved into phenolic biosynthesis [36]. Also, our results, and the evidence provided by other studies related to salt stress, suggested that phenolic compound content and antioxidant activities in plants may be decreased by salt stress [37–39]. The decrease of phenolic compounds is critically dependent on the salt sensitivity of plants in salt stress. Furthermore, we suggested that the increased accumulation of tyrosine and phenylalanine under treatments with excess Mg may be correlated with the downregulation of phenylpropanoid metabolism, which utilizes aromatic amino acids as metabolic precursors. Phenylalanine ammonia lyase (PAL) regulates the initial step of phenylpropanoid synthesis, and salinity stress affects the phenylpropanoid pathways, including PAL. *Lotus japonicus* exposed to salt stress exhibited reduced PAL activities and decreased levels of phenylpropanoids in the roots [40], consistent with the results of the present study. In addition, the low abundance of phenolic compounds may be attributed to the increase in the leaf chlorophyll content and the increased accumulation of carbohydrates resulting from Mg oversupply, which may limit the supply of the carbon skeleton to phenylpropanoid metabolism [41]. We examined bivariate correlations between the abundance patterns of significantly discriminant metabolites and plant phenotypes following treatment with excess Mg (Fig 3). The antioxidant assays revealed the strong positive correlations (*r* > 0.75) of leaf ABTS and FRAP with phenolic compounds, such as apigenin-7-*O*-diglucuronide, apigenin-6,8-diglucoside, luteolin-7-*O*-diglucuronide, luteolin-7-*O*-glucoside, caffeic acid, salvianolic acid C, and sagerinic acid. In this study, the leaf extracts of the plants under Mg oversupply (M7.5 and M10) presented relatively lower levels of flavonoids and cinnamic acid derivatives than those in the control group (Fig 2D), which may be attributed to their low antioxidant activities (Fig 1D–1F). High salinity can result in secondary stress conditions, including oxidative stress [6, 7]. It has previously been shown that secondary metabolites, such as phenolic compounds and flavonoids, act as major antioxidants, and their accumulation in plants reduces the oxidative damage caused by abiotic stress conditions, either directly or indirectly [42]. However, the low flavonoid and cinnamic acid derivative levels observed in the *P*. *frutescens* leaves treated with excess Mg in the present study are not in agreement with the previous observation. We hypothesize that high cellular Mg levels can lead to the substantial accumulation of soluble sugars, particularly hexoses, and amino acids to prevent osmotic stress, resulting in low levels of antioxidants, such as phenolic compounds [27, 41, 43]. We emphasize future multi-omics studies probing the biochemical pathways involved in plant’s stress response with compatible solutes and antioxidants compounds under high salinity conditions including the metal salts.

We noted a linear increase in the relative abundance of chlorins (pheophorbide b, pheophorbide a, and hydroxypheophorbide a) following treatment with excess Mg. Furthermore, the relatively low abundance of oxoglutaric acid coupled with the high levels of glutamic acid might have resulted in the increased production of chlorophyll pigments. The first step in chlorophyll biosynthesis is catalyzed by magnesium chelatase. The magnesium-rich form of the chelatase is a more effective catalyst of the chelation reaction. Magnesium chelatase activity is very sensitive to free magnesium concentrations [44]. In this study, the leaf chlorophyll content was higher in the *P*. *frutescens* leaves under Mg oversupply, as shown in Fig 1C. Generally, as chlorophyll degrades during leaf senescence, it is hydrolyzed into phytol and porphyrin moieties [45]. We further noted a positive correlation between the leaf chlorophyll content and most amino acids, carbohydrates, and chlorins (Fig 3). We further observed positive correlations between the leaf chlorophyll content and the relative chlorin abundance. Dephytylated chlorophylls (chlorophyllides and pheophorbides) are chlorins that act as intermediates in chlorophyll catabolism in green tissues [46]. We hypothesize that, under excess Mg, the chlorophyll degradation pathway was promoted in the *P*. *frutescens* leaves, resulting in high levels of chlorophyll pathway intermediates (pheophorbide a, pheophorbide b, and hydroxypheophorbide a) coupled with the chlorophyll degradation.

## Conclusions

Magnesium, which is an essential element, performs numerous metabolic functions; thus, it is widely applied to enhance the quality of agricultural produce. However, the overuse of Mg in greenhouses results in salt accumulation in the soil, which adversely affects crop growth. In the present study, we hypothesized that high levels of Mg may markedly alter the growth and quality of *P*. *frutescens* leaves. To verify this hypothesis, we examined the differential levels of metabolites, plant phenotypes, and antioxidant activities under Mg oversupply. We further attempted to correlate the metabolic profiles with the growth (plant height and total fresh weight of the shoot) and biochemical phenotypes (leaf chlorophyll content and antioxidant assays). We found that carbohydrate and amino acid metabolisms were upregulated under excess Mg, whereas phenylpropanoid metabolism was downregulated. These metabolic alterations were associated with lower growth and antioxidant activities and a higher chlorophyll content in the *P*. *frutescens* leaves. The study demonstrates the metabolic plasticity in plants, which allows them to circumvent the high salt stress, including Mg stress, caused by the excessive use of chemical fertilizers during greenhouse cultivation. This study improves our global understanding of the complex metabolic mechanisms occurring in plants under salt stress; however, a multi-omics approach, combining the “gene–protein–metabolite” interactions, is required for a more comprehensive understanding of plant stress responses.

## Supporting information

S1 FigPrincipal components analysis score (PCA) plots of *Perilla frutescens* leaves under magnesium oversupply derived from GC-TOF-MS (A) and UHPLC-LTQ-Orbitrap-MS/MS (B).Symbols indicated: ▲, Control; ▲, M7.5; ▲, M10. M7.5, magnesium supply at 7.5 times the control; M10, magnesium supply at 10 times the control. Three different plants were used as biological replicates.(DOCX)

S1 Table Primary metabolites identified by GC-TOF-MS in *Perilla frutescens* leaves under magnesium oversupply. * Differential metabolites were selected based on the VIP value (>0.7) and *p*-value (<0.05) from the orthogonal projection to latent structures-discriminant analysis model in Fig 2A. ^a^ Retention time; ^b^ Trimethylsilyl; ^c^ Identification. MS, mass spectrum compared with the National Institute of Standards and Technology (NIST) database and in-house libraries; STD, mass spectrum consistent with that of the standard compounds.
(DOCX)

S2 Table Secondary metabolites in *Perilla frutescens* leaves under magnesium oversupply derived from the UHPLC-LTQ-Orbitrap-MS/MS and UHPLC-LTQ-IT-MS/MS analyses. * Differential metabolites were selected based on the VIP value (>0.7) and *p*-value (<0.05) from the orthogonal projection to latent structures-discriminant analysis model in Fig 2B. ^a^ Retention time; ^b^ Molecular weight; ^c^ Reference; ^d^ In-house library; ^e^ Shoulder.
(DOCX)
