# Supplementary material for: Metabolomic response of Perilla frutescens leaves, an edible-medicinal herb, to acclimatize magnesium oversupply
Source: PLoS One. 2020 Jul 29;15(7):e0236813. doi: 10.1371/journal.pone.0236813 (PMC7390343; doi:10.1371/journal.pone.0236813)
Supplement: S1 Fig — Symbols indicated: ▲, Control; ▲, M7.5; ▲, M10. M7.5, magnesium supply at 7.5 times the control; M10, magnesium supply at 10 times the control. Three different plants were used as biological replicates. (DOCX) [file pone.0236813.s001.docx]

**
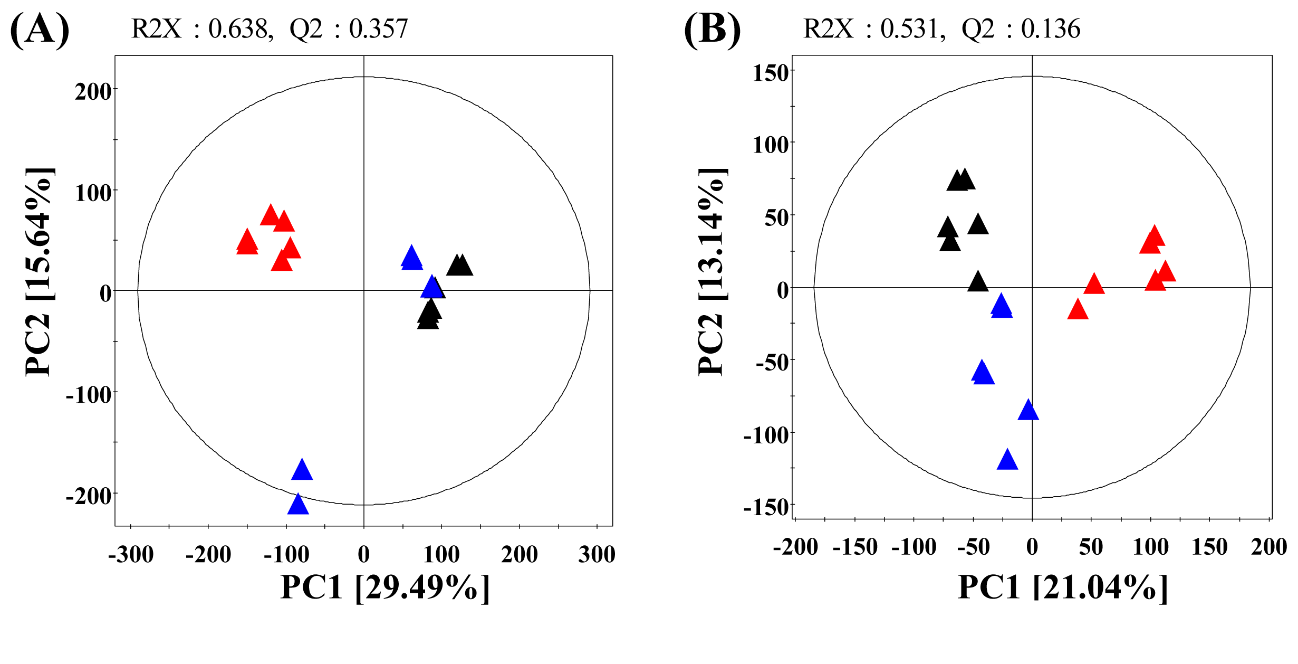
**

**S1 Figure. Principal components analysis (PCA) score plots of *Perilla frutescens* leaves under magnesium oversupply derived from GC-TOF-MS (A) and UHPLC-LTQ-Orbitrap-MS/MS (B).** Symbols indicated: ▲, Control; ▲, M7.5; ▲, M10. M7.5, magnesium supply at 7.5 times the control; M10, magnesium supply at 10 times the control. Three different plants were used as biological replicates.
