# Supplementary material for: Metabolomic response of Perilla frutescens leaves, an edible-medicinal herb, to acclimatize magnesium oversupply
Source: PLoS One. 2020 Jul 29;15(7):e0236813. doi: 10.1371/journal.pone.0236813 (PMC7390343; doi:10.1371/journal.pone.0236813)
Supplement: S1 Table — * Differential metabolites were selected based on the VIP value (>0.7) and p-value (<0.05) from the orthogonal projection to latent structures-discriminant analysis model in Fig 2A. a Retention time; b Trimethylsilyl; c Identification. MS, mass spectrum compared with the National Institute of Standards and Technology (NIST) database and in-house libraries; STD, mass spectrum consistent with that of the standard compounds. (DOCX) [file pone.0236813.s002.docx]

**S1 Table.** Primary metabolites identified by GC-TOF-MS in *Perilla frutescens* leaves under magnesium oversupply.

| **NO.** | **Tentative identification** | **GC-TOF-MS** | | | | |
| --- | --- | --- | --- | --- | --- | --- |
|  |  | **RT(min) ^a^** | **Mass** | **Mass Fragment** | **TMS ^b^** | **ID ^c^** |
| *Amino acids* | | | | |  |  |
| 1 | Alanine | 5.46 | 116 | 116 147 190 100 59 | 2 | STD |
| 2 | Valine | 6.61 | 144 | 218 100 283 159 114 | 2 | STD |
| 3 | Glycine* | 7.52 | 174 | 174 133 100 117 133 | 3 | STD |
| 4 | Serine* | 8.01 | 204 | 204 59 218 116 59 | 3 | STD |
| 5 | Threonine* | 8.26 | 219 | 117 176 219 291 320 | 3 | STD |
| 6 | Aspartic acid | 9.39 | 232 | 202 232 59 117 163 | 3 | STD |
| 7 | GABA* | 9.47 | 174 | 86 100 117 133 174 | 3 | STD |
| 8 | Glutamic acid | 10.16 | 246 | 218 204 174 156 128 | 3 | STD |
| 9 | Phenylalanine | 10.26 | 192 | 218 192 65 100 176 | 2 | STD |
| 10 | Tyrosine | 12.5 | 218 | 179 218 280 354 382 | 3 | STD |
| 11 | Tryptophan* | 14.29 | 202 | 202 231 291 348 173 | 2 | STD |
| *Organic acids* | |  |  |  |  |  |
| 12 | Lactic acid* | 5.02 | 234 | 191 219 88 66 118 | 2 | STD |
| 13 | Succinic acid | 7.54 | 247 | 247 218 172 129 116 | 2 | STD |
| 14 | Malic acid | 9.12 | 233 | 189 233 265 307 101 | 3 | STD |
| 15 | Citric acid | 11.69 | 273 | 273 257 211 183 99 | 4 | STD |
| 16 | Quinic acid | 12.02 | 345 | 255 345 419 191 204 | 5 | STD |
| 17 | Oxoglutaric acid* | 11.94 | 173 | 173 157 153 159 116 | 2 | STD |
| *Carbohydrates* | |  |  |  |  |  |
| 18 | Glycerol* | 7.2 | 177 | 103 117 177 205 218 | 3 | STD |
| 19 | Glyceric acid* | 7.74 | 189 | 189 217 292 130 190 | 3 | STD |
| 20 | Threonic acid | 9.75 | 292 | 117 133 189 205 220 | 4 | STD |
| 21 | Xylose* | 10.69 | 217 | 217 189 307 89 104 | 4 | STD |
| 22 | Xylitol* | 10.84 | 307 | 191 217 243 277 319 | 5 | STD |
| 23 | Adonitol* | 11.18 | 319 | 189 217 319 59 129 | 5 | MS |
| 24 | Fructose* | 12.13 | 217 | 217 277 307 364 129 | 5 | STD |
| 25 | Galactose | 12.33 | 160 | 205 291 319 160 117 | 5 | STD |
| 26 | Glucose* | 12.44 | 129 | 129 189 205 319 320 | 5 | STD |
| 27 | Gluconic acid* | 12.97 | 292 | 189 217 292 319 333 | 5 | STD |
| 28 | *myo-Inositol* | 13.558 | 191 | 191 217 221 246 265 | 6 | STD |
| 29 | Sucrose* | 16.66 | 361 | 437 361 319 271 215 | 8 | STD |
| 30 | Ononitol | 16.98 | 259 | 349 319 259 217 204 | 5 | MS |
| 31 | Maltose* | 17.81 | 204 | 361 319 271 204 59 | 8 | STD |
| *Fatty acids* | |  |  |  |  |  |
| 32 | Palmitic acid | 13.07 | 117 | 117 132 201 285 313 | 1 | STD |
| 33 | Linolenic acid | 14.15 | 108 | 173 129 108 67 335 | 1 | STD |
| 34 | Stearic acid | 14.25 | 132 | 341 201 117 129 132 | 1 | STD |
| 35 | Oleamide | 15.24 | 144 | 184 198 226 338 55 | 1 | STD |
| 36 | 1-Monopalmitin | 16.14 | 371 | 371 239 218 203 129 | 2 | STD |
| *Others* | |  |  |  |  |  |
| 37 | Adenosine | 16.5 | 192 | 280 236 230 192 103 | 4 | STD |

* Differential metabolites were selected based on the VIP value (>0.7) and *p*-value (<0.05) from the orthogonal projection to latent structures-discriminant analysis model in Figure 2A. ^a^ Retention time; ^b^ Trimethylsilyl; ^c^ Identification. MS, mass spectrum compared with the National Institute of Standards and Technology (NIST) database and in-house libraries; STD, mass spectrum consistent with that of the standard compounds.
