# Supplementary material for: Metabolomic response of Perilla frutescens leaves, an edible-medicinal herb, to acclimatize magnesium oversupply
Source: PLoS One. 2020 Jul 29;15(7):e0236813. doi: 10.1371/journal.pone.0236813 (PMC7390343; doi:10.1371/journal.pone.0236813)
Supplement: S2 Table — * Differential metabolites were selected based on the VIP value (>0.7) and p-value (<0.05) from the orthogonal projection to latent structures-discriminant analysis model in Fig 2B. a Retention time; b Molecular weight; c Reference; d In-house library; e Shoulder. (DOCX) [file pone.0236813.s003.docx]

**S2 Table.** **Secondary metabolites in *Perilla frutescens* leaves under magnesium oversupply derived from the UHPLC-LTQ-Orbitrap-MS/MS and UHPLC-LTQ-IT-MS/MS analyses.**

| **No.** | **Tentative Metabolite** |  | **UHPLC-LTQ-Orbitrap-MS** | | | | | | | **UHPLC-LTQ-IT-MS** | **References** |
| --- | --- | --- | --- | --- | --- | --- | --- | --- | --- | --- | --- |
|  |  | **RT^a^ (min)** | **[M-H]^-^** | **[M+H]^+^** | **M.W.^b^** | **Molecular Formula** | **Delta ppm** | **MS^n^ fragments** | | **UV λ max (nm)** |  |
| ***Flavonoids*** | | | | | | | | | | | |
| 1 | Apigenin-7-O-diglucuronide* | 4.61 | 621.112 | 623.123 | 622 | C27H27O17 | -1.181 |  | 623, 447, 271, 229, 203, 153, 163, 121 | 282, 325 | Ref ^c^ [S1] |
| 2 | Apigenin-6,8-di-C-glucoside* | 4.14 | 593.152 | 595.165 | 594 | C27H31O15 | -0.565 |  | 595, 577, 559, 529, 457, 439, 409, 379 | ─ | Ref [S6] |
| 3 | Apigenin-7-O-glucuronide* | 5.12 | 445.079 | 447.091 | 446 | C21H19O11 | -0.478 |  | 447, 271, 229, 203, 153, 111, 67 | 235, 304, 338 | Ref [S1] |
| 4 | Liquiritigenin* | 7.36 | 255.067 | 257.08 | 256 | C15H13O4 | -1.655 |  | 257, 239, 215, 173, 153, 131, 111 | 219, 330 | Ref [S2] |
| 5 | Luteolin-7-O-diglucuronide* | 4.38 | 637.106 | 639.121 | 638 | C27H27O18 | -1.8 |  | 637, 351, 285, 193, 175, 113 | ─ | LIB^d^ |
| 6 | Luteolin-7-O-glucoside* | 4.91 | 447.151 | 449.108 | 448 | C21H21O11 | -0.307 |  | 447, 357, 285, 181, | 285,322(sh)^e^ | Ref [S1] |
| ***Cinnamic acid derivatives*** | | | | | | | | | | | |
| 7 | Caffeic acid* | 3.7 | 179.036 | 181.049 | 180 | C9H9O4 | -0.637 |  | 179, 161, 135 | ─ | Ref [S1] |
| 8 | Salvianolic acid C* | 5.4 | 491.099 | 493.114 | 492 | C26H21O10 | -1.811 |  | 491, 447, 311, 267, 283, 255, 174 | 327 | LIB |
| 9 | 3,4-Dimethoxycinnamic acid* | 4.64 | 207.066 | 209.081 | 208 | C11H13O4 | -1.126 |  | 207, 179, 161, 135 | 298(sh), 326(sh) | LIB |
| ***Terpenoids*** | | | | | | | | | | | |
| 10 | Tormentic acid* | 4.81 | 487.344 | 489.356 | 488 | C30H47O5 | 4.758 |  | 487, 469, 423, 405, 393 | 296(sh), 327(sh) | Ref [S1] |
| 11 | Corosolic acid* | 4.85 | 471.35 | 473.365 | 472 | C30H47O4 | 4.866 |  | 471, 452, 423, 407, 393, 378, 390, 376, 350 | 221, 294(sh), 326 | Ref [S3] |
| 12 | Ursolic acid | 5.3 | 455.353 | 457.366 | 456 | C30H47O3 | 3.246 |  | 455, 407, 391, 377, 363, 335, 305, 251 | 226 | LIB, Ref [S4, S5] |
| ***Chlorins*** | | | | | | | | | | | |
| 13 | Pheophorbide b* | 10.19 | 605.242 | 607.256 | 606 | C35H35N4O6 | -2.686 |  | 607, 579, 547, 519, 475, 447, 419 | ─ | Ref [S6] |
| 14 | Hydroxy pheophorbide a* | 10.75 | 607.256 | 609.268 | 608 | C35H37N4O6 | -4.122 |  | 609, 591, 559, 459, 503, 487, 475, 459 | 226 | Ref [S7] |
| 15 | Pheophorbide a* | 10.91 | 591.26 | 593.273 | 592 | C35H37N4O5 | -3.956 |  | 593, 565, 533, 461 | ─ | Ref [S7] |
| ***Others*** | | | | | | | | | | | |
| 16 | Sagerinic acid | 8.41 | 719.16 | 721.175 | 720 | C36H31O16 | 2.015 |  | 719, 359, 223, 197, 161, 133 | 324 | Ref [S1], LIB |

* Differential metabolites were selected based on the VIP value (>0.7) and *p*-value (<0.05) from the orthogonal projection to latent structures-discriminant analysis model in Figure 2B. ^a^ Retention time; ^b^ Molecular weight; ^c^ Reference; ^d^ In-house library; ^e^ Shoulder.

**Supplementary references**

1. Lee YH, Kim B, Kim S, Kim MS, Kim H, Hwang SR, et al. Characterization of metabolite profiles from the leaves of green perilla (*Perilla frutescens*) by ultra high performance liquid chromatography coupled with electrospray ionization quadrupole time-of-flight mass spectrometry and screening for their antioxidant properties. J Food Drug Anal. 2017;25:776-788.
2. Ristivojević P, Trifković J, Gašić U, Andrić F, Nedić N, Tešić Ž, et al. Ultrahigh‐performance liquid chromatography and mass spectrometry (UHPLC–LTQ/Orbitrap/MS/MS) study of phenolic profile of Serbian poplar type propolis. Phytochem Anal. 2015;26:127-136.
3. Cao J, Peng LQ, Xu JJ. Microcrystalline cellulose based matrix solid phase dispersion microextration for isomeric triterpenoid acids in loquat leaves by ultrahigh-performance liquid chromatography and quadrupole time-of-flight mass spectrometry. J Chromatogr A 2016;1472:16-26.
4. Singh A, Bajpai V, Kumar S, Kumar B, Srivastava M, Rameshkumar KB. Comparative profiling of phenolic compounds from different plant parts of six Terminalia species by liquid chromatography–tandem mass spectrometry with chemometric analysis. Ind Crop Prod. 2016;87:236-246.
5. Chen Q, Zhang Y, Zhang W, Chen Z. Identification and quantification of oleanolic acid and ursolic acid in Chinese herbs by liquid chromatography–ion trap mass spectrometry. Biomed Chromatogr. 2011;25:1381-1388.
6. Zheng GD, Zhou P, Yang H, Li YS, Li P, Liu EH. Rapid resolution liquid chromatography–electrospray ionisation tandem mass spectrometry method for identification of chemical constituents in Citri Reticulatae Pericarpium. Food Chem. 2013;136:604-611.
7. Mohn T, Plitzko I, Hamburger M. A comprehensive metabolite profiling of *Isatis tinctoria* leaf extracts. Phytochemistry. 2009;70:924-934.
